# A model of contributors to a trusting patient-physician relationship: a critical review using a systematic search strategy

**DOI:** 10.1186/s12875-024-02435-z

**Published:** 2024-06-01

**Authors:** Seraina Petra Lerch, Rahel Hänggi, Yara Bussmann, Andrea Lörwald

**Affiliations:** 1 Faculty of Behavioural and Cultural Studies, Ruprecht Karls-University, Heidelberg, Germany; 2 University Hospital of Old Age Psychiatry and Psychotherapy, University of Bern, Bern, Switzerland; 3 Institute of Medical Psychology, Heidelberg University Hospital, Bergheimer Str. 20, DE-69115 Heidelberg, Germany; 4 Institute for Medical Education, Department for Assessment and Evaluation, University of Bern, Bern, Switzerland

**Keywords:** Patient trust, Patient-physician relationship, Communication, Health education, Theory of trust

## Abstract

**Background:**

The lack of trust between patients and physicians has a variety of negative consequences. There are several theories concerning how interpersonal trust is built, and different studies have investigated trust between patients and physicians that have identified single factors as contributors to trust. However, all possible contributors to a trusting patient-physician relationship remain unclear. This review synthesizes current knowledge regarding patient-physician trust and integrates contributors to trust into a model.

**Methods:**

A systematic search was conducted using the databases MEDLINE (Ovid), Embase (Ovid), PsycINFO (Ovid), and Eric (Ovid). We ran simultaneous searches for a combination of the phrases: patient-physician relationship (or synonyms) and trust or psychological safety. Six-hundred and twenty-five abstracts were identified and screened using pre-defined criteria and later underwent full-text article screening. We identified contributors to trust in the eligible articles and critically assessed whether they were modifiable.

**Results:**

Forty-five articles were included in the review. Patient-centered factors that contributed modifiable promoters of trust included psychological factors, levels of health education and literacy, and the social environment. Physician-centered factors that added to a trusting patient-physician relationship included competence, communication, interest in the patient, caring, the provisioning of health education, and professionalism. The patient-physician alliance, time spent together, and shared decision-making also contributed to trusting relationships between patients and physicians. External contributors included institutional factors, how payments are made, and additional healthcare services.

**Discussion:**

Our model summarized modifiable contributors to a trusting patient-physician relationship. We found that providing sufficient time during patient-physician encounters, ensuring continuity of care, and fostering health education are promising starting points for improving trust between patients and physicians. Future research should evaluate the effectiveness of interventions that address multiple modifiable contributors to a trusting patient-physician relationship.

**Supplementary Information:**

The online version contains supplementary material available at 10.1186/s12875-024-02435-z.

## Introduction

Trust, as a cornerstone of human relationships, applies to the patient-physician relationship. Relationship building is a basic skill for the medical professional [1, 2]. There is evidence that trust between patients and doctors has a positive effect and, if trust is missing, leads to potentially negative consequences. A meta-analysis confirmed that trust was positively associated with improved health outcomes [3] in, for example, diabetes [4], cancer [5], and human immunodeficiency virus infections (HIV infections) [6]. Trust also increases positive behavioral outcomes in patients [7], such as treatment adherence [8, 9]. In contrast, low trust in physicians has been shown to negatively affect various patient health outcomes [4, 6, 10–14]. Economically, if trust in physicians is missing, it has adverse financial effects on healthcare systems [15]. Furthermore, a physician may be more likely to incur complaints when trusting relationships with patients are lacking [16].

In medicine, trust can be understood as being social or interpersonal [17, 18]. Social trust refers to individuals’ trust in institutions or systems, such as the healthcare system or physicians in general, while interpersonal trust refers to the trust between two individuals [18, 19]. Social trust is believed to affect interpersonal trust in medical settings [17, 18]. There are various theories of trust from different disciplines [20–23]. However, the most prominent interpersonal trust theory in psychology (and applied in medical settings) is from Mayer et al., who defined trust as the willingness of an individual to be vulnerable to the actions of another based on the expectation that the other will perform a particular action important to the trustor, irrespective of the ability to monitor or control the other party [24]. Their theory of interpersonal trust suggests that benevolence, integrity, ability, propensity to trust, and perceived risk are components of a trust relationship [24]. When applied to the patient-physician relationship, the physician’s ability, integrity, and benevolence act as contributors. At the same time, a patient’s propensity to trust—their willingness to trust others—and the perceived risk they take when trusting a physician are also important factors. However, the reality is likely more complex, and there are probably more contributors to a trusting patient-physician relationship than the theory proposes. While different evidence-based studies have investigated the patient-physician trust relationship, to our knowledge, there has been no synthesis of all the evidence-based contributors to the relationship. In 2000, there was a call for an empirical conceptualization of trust. Rather than single theories used to explain interpersonal patient-physician trust or studies investigating isolated contributors of trust, the idea was to synthesize empirical evidence concerning how patient-physician trust can evolve into a model [19]. A recent review on trust in the medical field has renewed the need for such an empirical conceptualization of patient-physician trust [25]. Therefore, this study aimed to summarize the empirical evidence, identify the contributors to a trusting patient-physician relationship, and integrate them into a model. This model can then be used to identify potential approaches and leverage points to improve patient-physician trust. The two main research questions were:

1. Which factors contribute to a trusting patient-physician relationship?
2. Which of these factors can act as potential leverage points to improve the patient-physician relationship?

In addition, we critically assessed contributors based on how they are already implemented in healthcare systems and medical education.

## Methods

As the research questions were too broad for a systematic or scoping review, a critical review with a systematic search approach was used to answer the first research question. Critical reviews focus on empirical research [26] to evaluate what is known about a specific topic and integrate it into a framework [26, 27]. They may use a systematic search strategy to integrate the strengths of systematic and critical reviews [27], including all relevant literature, to avoid biases.

### Search strategy

We searched the databases MEDLINE (Ovid), Embase (Ovid), PsycINFO (Ovid), and ERIC (Ovid) for a combination of terms (or synonyms) referring to the patient-physician relationship and trust or psychological safety. Database searches were run simultaneously as multifile searches in Ovid. For the results, Ovid’s de-duplicator was used. No study or clinical trial registries or online resources were searched. No experts were contacted, nor was a citation search conducted. A reproducible search for all of the databases is as follows:

Embase (1974 to January 13, 2022), ERIC (1965 to May 2021), Ovid MEDLINE(R) ALL (1946 to January 13, 2022), APA PsycInfo (1806 to January Week 1, 2022).

1. (patient* adj2 physician* adj2 (relation* or alliance or rapport)).ti, ab.
2. (trust* or psychological safety).ti, ab.
3. 1 and 2.
4. remove duplicates from 3.

We did not use any language, time period, study design, or other restrictions for the searches, and no search filters were used. The comprehensive literature search was run on January 13, 2022 and 630 articles were retrieved. An information specialist assisted in framing the research questions and provided information on the different types of reviews. Once a first draft of the search strategy was developed, multiple feedback rounds with the information specialist were conducted until the search strategy was finalized.

### Screening process

**Fig. 1:**
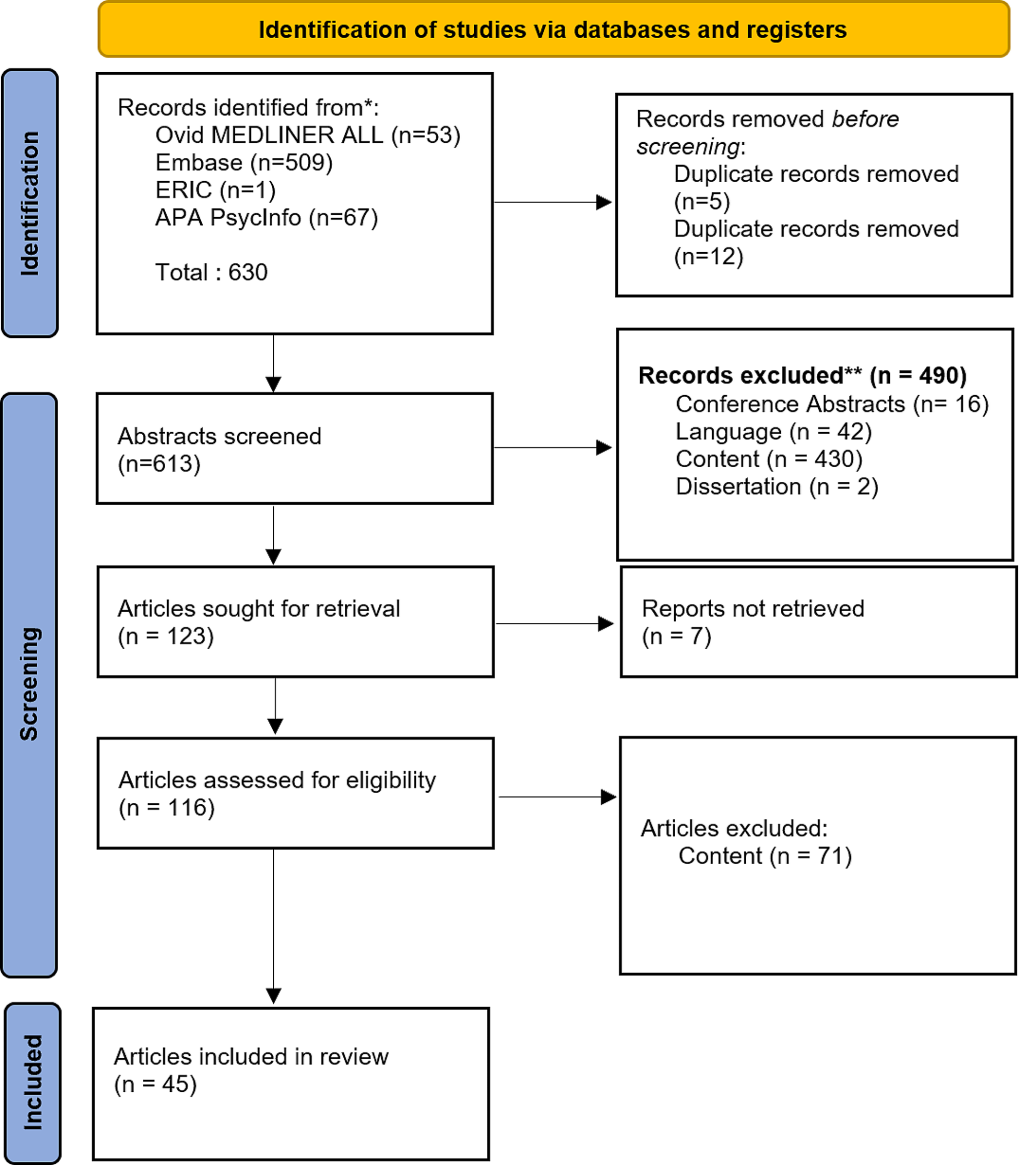
PRISMA study flowchart

Fifty-three records were retrieved from Ovid MEDLINER ALL, 509 from Embase, 1 from ERIC, and 67 from APA PsycInfo. In total, 630 records were found. As OVID’s de-duplication process did not identify all duplicates, any remaining duplicates were removed by SPL using EndNote’s duplicate identification strategy and a manual approach. After de-duplication, 613 articles remained, which were screened in two rounds. The first round was screened according to title and abstract. In the second round, 116 articles were evaluated for inclusion based on the full texts. SPL and RH did the screening, and AL decided when there were disagreements between SPL and RH. A study selection flowchart is shown in Fig. 1.

We included studies that reported factors contributing to a trusting relationship between patients and physicians and excluded those that only reported contributing factors between patients and health professionals other than physicians (or no contributors). We also included studies that explicitly measured trust between a patient and physician either quantitatively or qualitatively and excluded those with no measure of patient trust in physicians (e.g., only generalized patient trust or trust in other health professionals). We included quantitative, qualitative, and mixed methods papers and excluded dissertations and conference abstracts. Only articles in English and German were included.

### Data synthesis and categorization

We first extracted the contributors mentioned in the studies as this review focused on integrating contributors to a trusting patient-physician relationship into an overall model. Extracted data included information on the setting, patients, physicians, how trust was operationalized, and which factors had a positive, negative, or no effect on the relationship. Contributors were then categorized into patient-related, physician-related, context-related, or patient- and physician-related factors. Study sizes and methods of measurement were highlighted. The factors were then synthesized, and the modifiable ones were extracted and displayed in a model.

## Results

Forty-five heterogeneous studies reported factors contributing to a trusting patient-physician relationship. An overview of these studies, including the contributors to trust for each study, can be found in Appendix 1.

### Patient-related factors

Several patient-related contributors to a trusting relationship were investigated, sometimes with contrasting results from different studies. These included demographic characteristics (gender, marital status, age, ethnicity, birthplace, and country of residence), health condition, health education and literacy, socioeconomic status, religious beliefs, social environment, psychological factors, and the patient’s health condition and status.

Studies found better mental and physical health status tended to positively affect the relationship—although this result was mixed. In several studies, a good general health condition and better self-reported health status were associated with increased trust towards the physician [28–32]. However, other studies found no correlation between self-reported health status and trust. For specific health conditions, low-risk adults without chronic illnesses had higher trust in their physicians than adults with risk factors such as diabetes or high lipid levels [33–37]. Disease progression, including relapses and lack of improvement of a medical condition, was negatively associated with trust [38, 39], whereas a shorter duration of illness increased trust in the physician [29]. However, two studies found no connection between trust, disease duration [40], and healing [36].

Patient health education and literacy levels were found to promote a trusting patient-physician relationship, with higher health education [41] and literacy [42] levels contributing to trust and low health literacy [43] hindering it.

Patient socioeconomic status, including occupation, employment, educational and income levels, and the presence or type of health insurance, were all potential contributors, with high (household) income and educational levels, having health insurance, and being employed positively related to trust; although, these findings were ambiguous. Religious belief was also associated with trust in physicians [31].

The social environment, including social support and the care experiences of family members, further contributed to a trusting patient-physician relationship. In particular, poor social support negatively influenced trust [43], as did dissatisfaction with the care of family members [44, 45].

The health locus of control was also associated with trust. This describes how a person views control of their health. An internal health locus of control suggests that the person sees oneself as controlling their health, whereas an external locus means that the person perceives external factors influencing their health.

Several patient psychological factors, including a propensity to trust, their coping mechanisms and attachment style, the health locus of control, and general trust in caregivers contributed to a trusting patient-physician relationship. Individuals who see powerful others as their health locus of control (i.e., believing other people, such as health professionals, can control their health) exhibited higher trust in physicians [46]. Poor coping styles hindered trust [43], while the willingness to reframe situations (a healthy coping style) added to a trusting patient-physician relationship [44]. For the most part, a general trust in doctors, caregivers, the healthcare system, or online health communities was associated with higher trust [47–49]. However, these findings were ambiguous regarding the propensity to trust. One study found that a patient’s propensity to trust predicted trust in their physician [50], although other studies did not find this connection [31, 36]. Table 1 summarizes all of the evidence concerning patient-related factors.

**Table 1:** Overview of Patient*-*Related Contributors to a Trusting Relationship

Tested contributor to a trusting patient-physician relationship	Evidence of a positive effect on a trusting patient-physician relationship	Evidence of a negative effect on a trusting patient-physician relationship	No effect on a trusting patient-physician relationship
Demographic characteristics			
Sex	**- Being female** • (Bonds et al., 2004; *small sample size, other statistical method*) [35] • (Hillen et al., 2011; *small sample size, qualitative analysis*) [51] **- Being male** • (Gopichandran et al., 2015; *large sample size, other statistical method)* [52]	**- Being male** • (Wang et al., 2018; *very large sample size, other statistical method)* [53] **- Being female** • (Benjamins, 2006; *large sample size, other statistical method)* [31]	**- Sex** • (Aloba et al., 2014; Bachinger et al., 2009; Marcinowicz et al., 2017; *small sample size, correlation)* [33, 34, 40] • (Baidya et al., 2014; Fiscella et al., 2004; Hamelin et al., 2012; Zhao et al., 2016; *small sample size, other statistical method)* [28, 54–56] • (Dong et al., 2014; medium sample size, correlation) [57] (Gupta et al., 2014, *large sample size, other statistical method)* [58] • (Kao et al., 1998; *medium sample size, other statistical method)* [59]
Marital status	--	--	**- Marital status** • (Aloba et al., 2014; *small sample size, correlation*) [40] • (Bonds et al., 2004; Zhao et al., 2016; *small sample size, other statistical method*) [35, 56] • (Gupta et al., 2014, *large sample size, other statistical method)* [58]
Age	**- Older age** • (Bachinger et al., 2009; Marcinowicz et al., 2017; *small sample size, correlation)* [33, 34] • (Benjamins, 2006; Blanch-Hartigan et al., 2019; O’Malley et al., 2002; Oguro et al., 2021; *large sample size, other statistical method)* [30, 31, 45, 60] • (Bonds et al., 2004; Fiscella et al., 2004; Zhao et al., 2012; *small sample size, other statistical method)* [28, 35, 56] • (Hillen et al., 2011; Cook et al., 2004; *small sample size, qualitative analysis)* [41, 51] • (Dong et al., 2014; *medium sample size, correlation)* [57] • (Mainous et al. 2001; *large sample size, correlation)* [61]	--	**- Age** • (Gopichandran et al., 2015; Gupta et al., 2014; *large sample size, other statistical method)* [58, 62] • (Hamelin et al., 2012; *small sample size, other statistical method)* [55] • (Kao et al., 1998; *medium sample size, other statistical method)* [59]
Culture/Race/ Ethnicity	**-Members of other races (not black or white)** • (Benjamins, 2006; *large sample size, other statistical method)* [31] **-Cultural differences** • (Cook et al., 2004; *small sample size, qualitative analysis)* [41] **-Bedouins had more trust compared to Jews** • (Kushnir et al., 2008; small sample size, other statistical method) [63] **White individuals** • (Rawaf, 2007; *large sample size, other statistical method)* [64]	**-Afro-American** • (Hillen et al., 2011; *small sample size, qualitative analysis)* [51]	**-Race** • (Gupta et al., 2014; *large sample size, other statistical method)* [58] • (Kao et al., 1998; *medium sample size, other statistical method)* [48]
Birthplace	--	--	**Birthplace** • (Dong et al., 2014; *medium sample size, correlation)* [57]
Country/Place of residence	--	--	**-Country of residence** • (Mainous et al., 2001; *large sample size, correlation)* [61] **-Place of residence** • (Marcinowicz et al., 2017; *small sample size, correlation)* [34] **-Urban vs. rural residency** • (Wang et al., 2018; *very large sample size, other statistical method* [53] • (Zhao et al., 2016; *small sample size, other statistical method)* [56]
Health condition (low risk)	**-Low risk adults** (compared to adults with diabetes or high lipid levels) • (Becker & Roblin, 2008; *very large sample size, other statistical method)* [37]	--	**-Type of psychiatric diagnosis** • (Aloba et al., 2014; *small sample size, correlation)* [40]
Disease progression	**-Duration of illness (shorter)** • (Kowalski et al., 2009; *very large sample size, other statistical method)* [29]	**-Cancer relapse** • (Mack & Kang, 2020; *small sample size, other statistical method)* [38] **-Lack of improvement of condition** • (Yang et al., 2021; *medium sample size, other statistical method)* [39] **-Experiences of adverse events such as unexpected diagnoses and procedures** • (Shoemaker & Smith, 2019; *medium sample size, other statistical method)* [65]	**-Duration of illness** • (Aloba et al., 2014; *small sample size, correlation)* [40] **-Duration of the healing process** (Kao et al., 1998; *medium sample size, other statistical method)* [59]
Mental health status	**-Healthy mental status** • (Fiscella et al., 2004; *small sample size, other statistical method)* [28]	**-Somatization** (Fiscella et al., 2004; *small sample size, other statistical method)* [28]	--
Good general health condition	**-Good general health condition** • (Fiscella et al., 2004; *small sample size, other statistical method)* [28]; • (Kowalski et al., 2009; *very large sample size, other statistical method)* [29]; • (O’Malley et al., 2002, *large sample size, other statistical method)* [30]	--	--
Illness history	**-Previous number of hospital admissions** • (Aloba et al., 2014; *small sample size, correlation)* [40]	**-Previous visits to the hospital** • (Wang et al., 2018; *very large sample size, other statistical method)* [53]	**-Place of last checkup** • (Baidya et al., 2014; *small sample size, other statistical method)* [54]
Self-reported health status	**-Better self-rated health** • (Benjamins, 2006; *large sample size, other statistical method)* [31] • (Nelms et al., 2014; *small sample size, other statistical method)* [32]	--	**-Self-reported physical health status** • (Bachinger et al., 2009; Marcinowicz et al., 2017; *small sample size, correlation)* [33, 34] • (Bonds et al., 2004; *small sample size, other statistical method)* [35] • (Kao et al., 1998; *medium sample size, other statistical method)* [59] **-Self-reported mental health status** • (Bachinger et al., 2009; *small sample size, correlation)* [33]
Health education and literacy	**-High level of health education** • (Cook et al., 2004; *small sample size, qualitative analysis)* [41] **-Patient being informed** • (Dehghan et al., 2018; *medium sample size, other statistical method)* [42]	**-Low health literacy** • (Gupta et al., 2014; *large sample size, other statistical method)* [58]	--
Socioeconomic status			
Occupation/ Employment level	**-Employed** • (Rawaf et al., 2007; *large sample size, other statistical method)* [64]	--	**-Type of occupation** • (Baidya et al., 2014; *small sample size, other statistical method)* [54] • (Wang et al., 2018; *very large sample size, other statistical method)* [53] **-Employment status** • (Gopichandran et al., 2015; Gupta et al., 2014; *large sample size, other statistical method)* [52, 58]
Income	**-High income** • (Benjamins, 2006; *large sample size, other statistical method)* [31] • (Zhao et al., 2016; *small sample size, other statistical method)* [56]	--	**Income** • (Gupta et al., 2014; *small sample size, other statistical method)* [58] • (Bonds et al., 2004; Hamelin et al., 2012; *small sample size, other statistical method)* [35, 55]
Household income	**-Presence of household income** • (Benjamins, 2006; *large sample size, other statistical method)* [31]	--	--
Geographic or financial access	--	--	**- Geographic or financial access** • (O’Malley & Forrest, 2002; *large sample size, other statistical method)* [30]
Health insurance	**-Presence of health insurance** • (Benjamins, 2006; *large sample size, other statistical method)* [31]	--	**-Type of insurance** • (Dong et al., 2014; *medium sample size, correlation)* [57] **-Basic medical insurance for urban employees & commercial medical insurance** • (Wang et al., 2018; *very large sample size, other statistical method)* [53]
Educational level	**- High level of education** • (Dong et al., 2014; *medium sample size, correlation)* [57] • (Rawaf et al., 2007; *large sample size, other statistical method)* [64] • (Zhao et al., 2016; *small sample size, other statistical method)* [56] • (Cook et al., 2004; *small sample size, qualitative analysis)* [41]	--	**- Educational level** • (Bachinger et al., 2009; Marcinowicz et al., 2017; *small sample size, correlation)* [33, 34] • (Baidya et al., 2014; Hamelin et al., 2012; *small sample size, other statistical methods)* [54, 55] • (Gupta et al., 2014; Oguro et al., 2021; *large sample size, other statistical method)* [45, 58]
Religious beliefs	**-Attending religious services and religious affiliation** (mainline Protestant, Catholic, and Jewish) • (Benjamins, 2006; *large sample size, other statistical method* [31]	**-Religious affiliation** (other than mainline Protestant, Catholic, or Jewish) • (Benjamins, 2006; *large sample size, other statistical method)* [31]	**-Strength of religious affiliation** • (Benjamins, 2006; *large sample size, other statistical method* [31]
Social environment			
Social support	--	**- Poor social support** • (Gupta et al., 2014; *large sample size, other statistical method)* [58]	--
Experience of care received by family members	--	**- Experiencing the treatment of family members and interactions with physicians in a negative way** • (Canavera, 2021; *small sample size, qualitative analysis)* [44] **- Dissatisfaction with family members’ care** • (Oguro et al., 2021; *large sample size, other statistical method)* [45]	--
Family members health locus of control	**-Family members, powerful others, and internal health locus of control** • (Brincks et al., 2010; *small sample size, other statistical method)* [46]	**-Family members, chance health locus of control** • (Brincks et al., 2010; *small sample size, other statistical method)* [46]	--
Psychological Factors	--	**-Overall dissatisfaction with current life status and a higher emphasis on personal health** • (Wang et al., 2018; *very large sample size, other statistical method)* [53]	--
Health locus of control	**-Powerful others (healthcare providers) health locus of control** • (Brincks et al., 2010; *small sample size, other statistical method* [46]	--	--
Attachment style		**- Insecure attachment style** • (Holwerda et al., 2013; *small sample size, other statistical method)* [66]	--
Coping	**-Willingness to reframe situations** • (Canavera, 2021; *small sample size, qualitative analysis*) [44]	**- Poor coping skills** • (Gupta et al., 2014; *large sample size, other statistical method)* [58, 67]	--
Propensity to trust	**-Patient’s overall trust** • (Wu et al., 2021; *very large sample size, other statistical method)* [50]	--	**-General trust in people** • (Benjamins, 2006; *large sample size, other statistical method)* [31] • (Kao et al., 1998; *medium sample size, other statistical method)* [59]
Trust in caregivers and the healthcare system	**-General trust in doctors, institutions and nurses** • (Bonds et al., 2004; *small sample size, other statistical method)* [35] **-Trust in managed care organizations** • (Kao et al., 1998; *medium sample size, other statistical method* [59] **-Interpersonal trust in peer-to-peer online health communities and the information that is provided and exchanged there** • (Audrain-Pontevia & Menvielle, 2018; *large sample size, other statistical method)* [49]	**-Dissatisfaction with a hospital’s general condition** • (Yang et al., 2021; *medium sample size, other statistical method)* [39]	**- How the admittance process is perceived** • (Kowalski et al., 2009; *very large sample size, other statistical method)* [29]

* 1–250 = small sample size; 251–500 = medium sample size; 501–2000 = large sample size; > 2000 = very large sample size; other statistical method: method other than qualitative or correlation analysis such as regression analysis

### Physician-related factors

Demographic characteristics, competence, communication, exploring, caring, provisioning health education, reputation, professionalism, and availability were investigated as potential contributors to a trusting patient-physician relationship.

Demographic characteristics of the physician, such as age and gender, did not contribute to a trusting relationship, although these findings were ambiguous.

Physician competency, including the perceived competence of the physician by the patient [41, 44, 51, 55, 68–70], the physician being up-to-date in their specialization [71], and having more years of experience [71] helped to build a trusting relationship with patients. Communication skills, including general communication skills [29, 38, 44, 52, 70, 72, 73], compassion, listening to the patient [41, 44, 52], as well as nonverbal behavior such as good eye contact, providing undivided attention, open body language, and smiling [41, 44, 52, 73] also enhanced the trust relationship as did patient-centered [63, 74, 75], comprehensive care [30].

Physicians exploring a patient’s disease and problems [69], illness experiences [28], and the context of the patient [44, 68] promoted a trusting relationship along with caring behavior [52, 70, 75] such as empathy [50, 76] and compassion [41, 44, 69].

Provisioning health education to the patient contributed to a trusting relationship [38, 41, 64, 68, 69]; however, one study did not find any association between these factors [71].

We did identify physician reputation [71] and the reputation of their medical specialty [28, 34, 39] as contributing to a trusting relationship. Moreover, different aspects of professionalism [71, 73], such as honesty [51, 55, 69] and availability [41], contributed to a trusting patient-physician relationship, while being disrespectful, arrogant, or cynical were negatively associated with trust [41, 73, 75]. These results are summarized in Table 2.

**Table 2:** Overview of Physician-Related Contributors to a Trusting Relationship

Tested contributor to a trusting patient-physician relationship	Evidence of a positive effect on a trusting patient-physician relationship	Evidence of a negative effect on a trusting patient-physician relationship	No effect on a trusting patient-physician relationship
Demographic characteristics			
Sex	**-Being female** • (Bonds et al., 2004; *small sample size, other statistical method)* [35]	--	**-Sex** • (Baidya et al., 2014; *small sample size, other statistical method)* [54] • (Blanch-Hartigan et al., 2019; *large sample size, other statistical method)* [60] • (Shaya et al., 2019; *small sample size, qualitative analysis)* [71]
Age	--	--	**-Physician age** • (Baidya et al., 2014; Fiscella et al., 2004; *small sample size, other statistical method)* [28, 54] • (Weng, 2008; *large sample size, other statistical method)* [76]
Competence/Experience			
Perceived competence by the patient	-**High competency perceived by the patient** • (Berry et al., 2008; *large sample size, other statistical method)* [68] • (Canavera, 2021; Cook et al., 2004; Hillen et al., 2011; Thom & Campbell, 1997; Wolfson & Lynch, 2021 *small sample size, quantitative analysis*) [41, 44, 51, 69, 70] • (Hamelin et al., 2012; *small sample size, other statistical method)* [55]	--	--
Being up-to-date	**-The physician being up-to-date** • (Shaya et al., 2019; *small sample size, qualitative analysis)* [71]	--	--
Years of residency/experience	**-More years of experience** • (Shaya et al., 2019; *small sample size, qualitative analysis* [71]	--	**-Years of residency training** • (Bonds et al., 2004; *small sample size, other statistical method*) [35]
Making major mistakes	--	**-Physician making a major mistake** • (Shaya et al., 2019; *small sample size, qualitative analysis)* [71]	--
Communication			
Communication skills	**-Clear explanations** • (Gopichandran et al., 2015, *large sample size, other statistical method)* [62] **-Communicating clearly and competently** • (Canavera, 2021; Thom & Campbell, 1997; Wolfson & Lynch, 2021; Hendren & Kumagi, 2019 *small sample size, qualitative analysis)* [44, 69, 70, 73] **-Physician facilitating communication** • (Kowalski et al., 2009; *very large sample size, other statistical method)* [29] **-High-quality communication** • (Mack & Kang, 2020; *small sample size, other statistical method)* [38]	**-Giving information in an insensitive manner** • (El Malla et al., 2013; *medium sample size, other statistical method)* [75]	**-Verbal uncertainty of the physician** (Blanch-Hartigan et al., 2019; *large sample size, other statistical method)* [60]
Listening	**- Compassionate and attentive listening** • (Canavera, 2021; Cook et al., 2004; *small sample size, quantitative analysis)* [41, 44] **-Listens patiently** • (Gopichandran et al., 2015, *large sample size, other statistical method)* [62]	--	--
Nonverbal behavior	**-Nonverbal behavior such as good eye contact, undivided attention, open body language, and smiling** • (Canavera, 2021; Cook et al., 2004; Hendren & Kumagi, 2021; *small sample size, quantitative analysis)* [41, 44, 73] • (Gopichandran et al., 2015; *large sample size, other statistical method)* [62]	**-Negative nonverbal behavior of high uncertainty** • (Blanch-Hartigan et al., 2019; *large sample size, other statistical method)* [60] **-No eye contact & sending nonverbal messages** • (Canavera, 2021; Cook et al., 2004; *small sample size, qualitative analysis)* [41, 44]	--
Patient-centered care	**-Patient-centered behavior** • (Hillen et al., 2011; *small sample size, qualitative analysis)* [51] • (Kushnir et al., 2008; *small sample size, correlation)* [63] • (El Malla et al., 2013; *medium sample size, other statistical method)* [75] **-Comprehensive care** • (0’Malley & Forest, 2020; *large sample size, other statistical method)* [30]	--	--
Exploration (understanding the patient’s context and experiences)	**-Physician understanding the patient’s context and experiences and thoroughly evaluating patient problems** • (Thom & Campbell, 1997; *small sample size, qualitative analysis)* [69] **-Physician wanting to know the patient** • (Berry et al., 2008; *large sample size, other statistical method)* [68] • Canavera, 2021; *small sample size, quantitative analysis)* [41] **-Exploring patient experience of disease and illness** • (Fiscella et al., 2004; *small sample size, other statistical method)* [28]	--	**-Personal involvement (**knows the family situation, knows the name of patient, treats the patient like family) • (Gopichandran et al., 2015, *large sample size, other statistical method* [62]
Empathy/Compassion/ Caring	**-Compassion** • (Canavera, 2021; Cook et al., 2004; Thom & Campbell, 1997; *small sample size, qualitative analysis)* [41, 44, 69] • Empathy (Wu et al., 2021; *very large sample size, other statistical method)* [50] • (Weng, 2008, *large sample size, other statistical method)* [76] **-Caring/Comfort** • (Gopichandran et al., 2015, *large sample size, other statistical method* [62] • (Wolfson & Lynch, 2021; *small sample size, other statistical method)* [70] • (El Malla et al., 2013; *medium sample size, other statistical method)* [75]	--	--
Providing health Education	**-Providing and explaining information** • (Cook et al., 2004; *small sample size, quantitative* analysis) [41] • (Mack & Kang, 2020; *small sample size, other statistical method)* [38] **-Experiences with provider** (more information/exchange about hypertension and its management) • (Rawaf & Kressin, 2007; *large sample size, other statistical method)* [64] **-Autonomy support** • (Berry et al., 2008; *large sample size, other statistical method)* [68] **-Physician offering all options for medical treatment** • (Thom & Campbell, 1997; *small sample size, qualitative analysis* [69]	**-Physician’s failure to provide adequate and clear explanations** • (Cook et al., 2004; *small sample size, quantitative analysis*) [41]	**- Educating patients** • (Shaya et al., 2019; *small sample size, qualitative analysis)* [71]
Reputation			
Reputation of the physician	-**Good reputation of the physician;** Physician being recommended by a family member or being a family member themselves, physician being featured in the media • (Shaya et al., 2019; *small sample size, qualitative analysis)* [71]	--	--
Reputation of medical specialty	**-Family practice** • (Fiscella et al., 2004; *small sample size, other statistical method)* [28] • (Marcinowicz et al., 2017; *small sample size, correlation)* [34] **-Trust in emergency physicians and cardiologists was higher (compared to pediatricians)** • (Yang et al., 2021; *medium sample size, other statistical method)* [39]	**-Trust in pediatricians was lower (compared to emergency physicians and cardiologists)** • (Yang et al., 2021; *medium sample size, other statistical method)* [39] **Practice Background** • (Baidya et al., 2014 [54]; *small sample size, other statistical method)*	--
Professionalism	**-Physician’s attire and hygiene** (professional attire) • (Shaya et al., 2019; *small sample size, qualitative analysis)* [71]	**-Negligence** • (Hendren & Kumagi, 2019; *small sample size, qualitative analysis)* [73]	--
Disrespectful, arrogant or cynical attitude	--	**-Disrespectful and arrogant attitude** • (El Malla et al., 2013; *medium sample size, other statistical method)* [75] • (Hendren & Kumagi, 2019; *small sample size, qualitative analysis)* [73] **-Lack of concern** (Hendren & Kumagi, 2019; *small sample size, qualitative analysis)* [73] **-Physicians’ failure to make patients feel respected** (Cook et al., 2004; *small sample size, quantitative analysis)* [41]	**-Cynicism** • (Kao et al., 1998; *medium sample size, other statistical method)* [59]
Honesty	**-Physician telling the truth about the medical condition of the patient** • (Hamelin et al., 2012; *small sample size, other statistical method)* [55] **-Honesty** • (Hillen et al., 2011; Thom & Campbell, 1997; *small sample size, qualitative analysis)* [51, 69]	--	--
Availability	**-Physician being available** • (Cook et al., 2004; *small sample size, quantitative analysis)* [41] • (Kowalski et al., 2009; *very large sample size, other statistical method*) [29]	**-Physician not being available** • (Cook et al., 2004; *small sample size, quantitative analysis)* [41]	

* 1–250 = small sample size; 251–500 = medium sample size; 501–2000 = large sample size; > 2000 = very large sample size; other statistical method: method other than qualitative or correlation analysis such as regression analysis

### Physician- and patient-related factors

Contributors related to the physician and patient were concordance, time spent together, the patient-physician alliance, and shared decision-making.

In relation to concordance, both gender and race were tested as promoters of trust; however, only gender concordance was identified as being a contributor [35, 41].

Time spent together included time spent in a single session, the overall time spent together, and the continuity of care. Most results indicated that more time spent together in a single session [28, 71, 73] (with the physician giving the patient enough time to explain the reason for the visit [77]) promoted trust, whereas physicians appearing rushed was a barrier to a trusting relationship [44]. If the duration of the relationship with the doctor was long-term [28, 36, 77], the patient had higher rates of follow-up visits [51] and more physician visits in general [37, 57]. Nevertheless, those findings were mixed, and not all studies found an association between the duration of a relationship with the doctor [40, 45] and the number of team visits [47, 48]. However, continuity of care [51] and continuity with one physician added to a trusting relationship [30].

Within the patient-physician alliance, alliances in shared decision-making [65] and having a good rapport [71] were found to enhance trust, while a patient’s perception of a physician’s distrust was a barrier [41]. Finding common ground [28] and shared identity [52] were tested but did not show any association with trust. In contrast, shared decision-making contributed to a trusting relationship that promoted trust in most studies [41, 42, 44]. These findings are summarized in Table 3.

**Table 3:** Overview of Patient-Physician Contributors to a Trusting Relationship

Tested contributor to a trusting patient-physician relationship	Evidence of a positive effect on a trusting patient-physician relationship	Evidence of a negative effect on a trusting patient-physician relationship	No effect on a trusting patient-physician relationship
Concordance	**-Gender concordance** • (Bonds et al., 2004; *small sample size, other statistical method)* [35] • (Cook et al., 2004, *small sample size, qualitative analysis)* [41]	--	**-Race concordance** (Bonds et al., 2004; *small sample size, other statistical method)* [35]
Time spent together			
Time spent together in a single session	**-More time spent with the physician (in a single session)** • (Fiscella et al., 2004; *small sample size, other statistical method)* [28] • (Shaya et al., 2019; *small sample size, qualitative analysis)* [71] **-Time** • (Hendren et al., 2019; *small sample size, qualitative analysis)* [73] **-Length of time with one’s regular physician** (longer = more trust) and the importance of seeing one’s regular physician every time • (Mainous et al., 2001; *large sample size, correlation)* [61] **-Physician giving the patient enough time to explain the reason for the visit** • (Hamelin et al., 2012; *small sample size, other statistical method)* [55]	**-Physician appears rushed** • (Canavera et al., 2021; *small sample size, qualitative analysis)* [44]	**-Time spent with the physician** (in a single session) • (Baidya et al., 2014; *small sample size, other statistical method)* [54]
Overall time spent together	**-Duration of the relationship with the physician** (longer = more trust) • (Fiscella et al., 2004; *small sample size, other statistical method)* [28] • (Kao et al., 1998; *medium sample size, other statistical method)* [59] • (Parchman et al., 2004; *very large sample size, other statistical method)* [77] **-High rate of patient follow-up visits** • (Weng et al., 2008; *large sample size, other statistical method)* [76] **-Amount of (team) visits** (more = more trust) • (Becker & Roblin, 2008; *very large sample size, other statistical method)* [37] • (Dong et al., 2014; *medium sample size; correlation)* [57]	--	**-Duration of relationship with the physician** • (Aloba et al., 2014; *small sample size, correlation)* [40] • (Oguro et al., 2021; *large sample size, other statistical method)* [45] **-Amount of (team) visits;** • (Bonds et al., 2004; *small sample size, other statistical method)* [35] • (Kao et al., 1998; *medium sample size, other statistical method)* [59]
Continuity of care	**-Previous care by a resident while hospitalized** • (Bonds et al., 2014; *small sample size, qualitative analysis)* [35] **-Continuity of care** • (Hillen et al., 2011; *small sample size, qualitative analysis)* [51] • (Mainous et al., 2001; *large sample size, correlation)* [61] **-Continuity with one physician** • (O’Malley et al., 2002; *large sample size, other statistical method)* [30]	--	--
Patient-physician alliance	**-Patient-physician alliance in decision making** • (Shoemaker & Smith, 2019; *medium sample size, other statistical method)* [65] • Good rapport (Shaya et al., 2019; *small sample size, qualitative analysis)* [71]	**-Patients’ perception of physician distrust** • (Cook et al., 2004; *small sample size; qualitative analysis)* [41]	**-Finding common ground** • (Fiscella et al., 2004; *small sample size, other statistical method)* [28] **-Shared identity** • (Gopichandran et al., 2015; *large sample size, other statistical method)* [62]
Shared decision making	**-Shared decision making** • (Canavera et al., 2021; *small sample size, qualitative analysis)* [44] **-Patient participation in the decision-making process** • (Cook et al., 2004; *small sample size, qualitative analysis*) [41] • (Dehghan et al., 2018; *medium sample size, other statistical method)* [42]		**-Patient participation in the decision-making process** • (Kao et al., 1998; *medium sample size, other statistical method)* [59]

* 1–250 = small sample size; 251–500 = medium sample size; 501–2000 = large sample size; > 2000 = very large sample size; other statistical method: method other than qualitative or correlation analysis such as regression analysis

#### Context-related factors

Context-related factors such as practice/institution, physician payments, and additional healthcare services were investigated as potential contributors to trusting relationships.

Most aspects of the practice or the institution were found to contribute to a trusting relationship, with easy accessibility [30] to the practice and a good reputation [71] promoting trust, while institutional betrayal [65] hindered it. The atmosphere of the practice also mattered. A good practice or organizational climate added to a trusting relationship [35], whereas perceived chaos hampered it [29]. Patients having enough physician choice also added to a trusting relationship [48], while managed care settings contributed to mistrust [41]. Inpatient settings enhanced trust compared to outpatient settings [59]. Regarding payments, situations where patients do not know how the physician is paid or the physician is paid by the number of office visits rather than a fixed salary [30] contributed to a trusting relationship. In contrast, public disclosure of payments was negatively associated with trust [78]. Additional health services such as addiction consultations [79], preventive services [77], and the coordination of specialty care [30] also contributed to patient-physician trust. These findings are summarized in Table 4.

**Table 4:** Overview of Context-Related Contributors to a Trusting Patient-Physician Relationship

Tested contributor to a trusting patient-physician relationship	Evidence of a positive effect on a trusting patient-physician relationship	Evidence of a negative effect on a trusting patient-physician relationship	No effect on a trusting patient-physician relationship
Practice/Institution	**- Inpatient setting** • (compared to outpatient) (Wang et al., 2018; very *large sample size, other statistical method)* [53]	**-Departments (**medical, surgical, pediatrics, gynecology and obstetrics) • (Wang et al., 2018; *very large sample size, other statistical method)* [53]	**-Practice type** • (Baidya et al., 2014; *small sample size, other statistical method)* [54] **-New rural cooperative medical system** • (Wang et al., 2018; *very large sample size, other statistical method)* [53]
Accessibility	**-Organizational accessibility of the practice** • (O’Malley et al., 2002; *large sample size, other statistical method)* [30]	--	--
Reputation	**-Good reputation of the practice** • (Shaya et al., 2019; *small sample size, qualitative analysis)* [71]	--	--
Institutional betrayal	--	**- Institutional betrayal** • (Shoemaker & Smith,2019; *medium sample size, other statistical method)* [65]	--
Practice/ organizational climate	**- Good practice climate** • (Becker & Roblin, 2008; *very large sample size, other statistical method)* [37]	**-Perceived chaos** • (Kowalski et al., 2009; *very large sample size, other statistical method)* [29]	--
Choice of physician	**-Having enough choice of physicians** • (Kao et al., 2008; *medium sample size, other statistical method)* [59]	**-Managed care settings** • (Cook et al., 2004; *small sample size, qualitative analysis)* [41]	--
Payment	**-Physician is paid by the number of office visits** (rather than a fixed salary) • (Kao et al., 1998; *very large sample size, other statistical method)* [48] **-Patients not knowing how the physician is paid** • (Kao et al., 1998; *very large sample size, other statistical method)* [48]	**-Public disclosure of payments** (regardless of whether respondents knew their physicians had received payments) • (Kanter et al., 2019; *very large sample size, other statistical method)* [78]	--
Healthcare services	**-Addiction consultation services for patients** • (King et al., 2021; *medium sample size, other statistical method)* [79] **-Preventative service delivery** • (Parchman et al., 2004; *very large sample size, other statistical method)* [77] **-Coordination of specialty care services** • (O’Malley et al., 2004; *large sample size, other statistical method)* [30]	--	--

* 1–250 = small sample size; 251–500 = medium sample size; 501–2000 = large sample size; > 2000 = very large sample size; other statistical method: method other than qualitative or correlation analysis such as regression analysis

### Potential leverage points to improve a trusting relationship

**Fig. 2:**
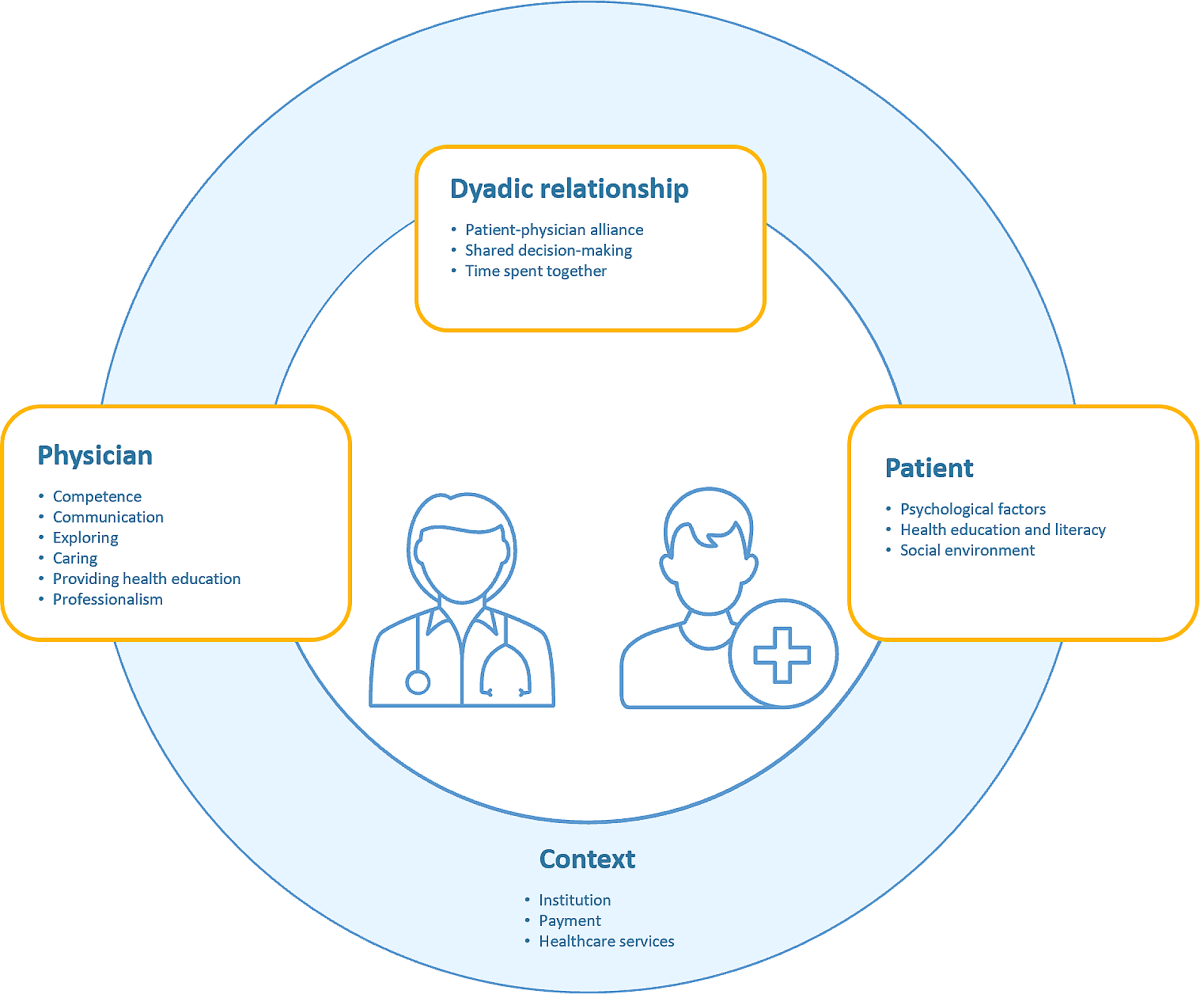
Model of contributors to a trusting patient-physician relationship

We integrated the modifiable contributors to a trusting patient-physician relationship from each conceptual group into a model and identified potential leverage points for improving the relationship (Fig. 2).

### Patient-centered leverage points

Within patient-centered factors, health education and literacy, the social environment, and psychological factors were modifiable. A patient who is better educated about health and can understand and use this education for themselves (health literacy) may form better trusting relationships with their physicians; thus, interventions should focus on improving health education and literacy. Patient psychological factors such as coping styles and health locus of control are other potential leverage points to increase trust within the relationship. The social environment, specifically receiving sufficient social support, was a further modifiable contributor to trust, indicating that targeted interventions should aim to improve patients’ social support systems.

### Physician-centered leverage points

We identified physicians’ competence, communication skills, exploring, caring, the provisioning of health education, and professionalism as modifiable contributors to a trusting patient-physician relationship. For competence, being up-to-date in the specialization and perceived as competent are leverage points that could increase trust. Communication skills, including verbal and nonverbal behavior, exploring patient health, and professionalism, can also be learned and are, hence, modifiable. Caring, including empathy and compassion, is a skill that can be increased through interventions and also used to increase trust. In addition, physicians can be taught how to provide health education, and specific material can be provided to them for health education, which is another potential leverage point.

### Patient and physician-centered leverage points

We identified shared decision-making, the patient-physician alliance, and time spent together as contributors that can be modified. Although time spent together and the continuity of care is context-dependent, awareness can be raised among physicians, and specific training can help the physician allow patients to explain the reason for their visit. Alliances and shared decision-making are skills taught during medical school: therefore, potential interventions already exist. Shared decision-making also includes healthcare professionals other than physicians. Therefore, one possible intervention strategy would be to foster interprofessional education and teamwork to support shared decision-making between patients and healthcare professionals.

### Context-dependent leverage points

The healthcare system, provisioning of additional healthcare services, transparency regarding physician payment, and characteristics of the practice or institution (e.g., keeping a good institutional climate and having mechanisms to prevent institutional betrayal) are modifiable contributors; however, these strongly depend on the specific country. Furthermore, only a few studies have investigated contributors to a trusting relationship within this conceptual group. Therefore, the list of context-dependent contributors may be limited.

## Discussion

We conducted a critical review with a systematic search strategy to identify evidence-based contributors to a trusting patient-physician relationship and integrated the modifiable contributors into a model. Our results confirm the existing theory of interpersonal trust [24], and, in line with this theory, we found that the physician’s caring (benevolence), competence and communication (ability), and professionalism (integrity) were contributors to a trusting patient-physician relationship. In addition, the physician’s exploring and provisioning of health education also contributed to a trusting relationship. We confirmed the importance of a patient’s propensity to trust as a psychological contributor and were able to add more psychological factors, including coping style and health locus of control. We further added the patient’s level of health education and literacy, and social environment as contributing factors and confirmed that, as the risk a patient must take concerning their health decreases, the easier it is for them to trust the physician. Our model further adds physician- and patient-related factors and the institutional context. The latter indicates the importance of including social trust in understanding interpersonal trust in medicine, as suggested by Mechanic [18]. One highly prominent factor was health education, which can be addressed by the physician, patient, and the context, which suggests that fostering health education is a promising intervention to increase trust.

### Patients

Patient psychological factors such as coping styles and health locus of control are modifiable contributors to a trusting relationship. Previous studies have shown that coping styles can be improved for chronically ill patients [80], while other interventions can address a patient’s health locus of control and improve their social support systems. Furthermore, social support interventions have been shown to be effective in patients with different diseases [81–83]. Health education could be addressed through e-learning and by provisioning self-help groups that exchange ideas about diseases [84] with educational tools and teaching materials [85]. However, these interventions are system-related as the healthcare system must offer those interventions.

### Medical education

Most physician-centered modifiable contributors to a trusting relationship fall under the scope of medical education. Competence is acquired and addressed through university education, graduate school, and continuing education. Communication skills are taught in medical education courses, and professionalism is addressed as a CanMED role [86]. Exploration is an important skill that is already part of communication curriculums [87] and is based on the common-sense model of illness [88]. Physicians can be taught to provide health education [89]; however, it is a skill that medical students find difficult to achieve [90]. Further intervention possibilities could address a physician’s ability to express compassion and empathy. A recent review summarized educational methods used to address medical student empathy [91], with simulation training shown to be an effective tool [92].

A practical example that implements the described practices can be found in the Presence 5 project, which teaches physicians to better listen to patients, explore their story and emotions, and connect with them. These teachings have had positive effects on the physicians’ attitude, compassion, communication, and exploring behavior [93, 94].

### Patient- and physician-related factors

As with physician-related contributors to trust, patient- and physician-related promoters of trust could be addressed through medical education. Building an alliance with patients and learning about shared decision-making are skills taught in medical school [95]. The physician can also be made aware that spending sufficient time with a patient is relevant to building trust; however, the ability to modify this contributor is dependent on the healthcare and billing system.

### Context-dependent contributors

We found that a transparent billing system and institution-related contributors such as reputation, medical practice atmosphere, accessibility, and additional healthcare services contributed to a trusting patient-physician relationship. A recent discussion on making health care more accessible can be found in Gupta et al. [96].

One healthcare system that addresses many of these factors is Canada’s patient-centered model: ‘the patient’s medical home.’ Under this model, patients can choose a physician they feel comfortable with and who will continuously manage their health care over their lifespan. Each physician is surrounded by a team that considers the patient’s situation and may provide additional healthcare services when needed. This model ensures that each patient receives comprehensive and accessible care that provides sufficient time with the physician and guarantees continuity of care [https://patientsmedicalhome.ca/, 97]. Over the long term, patient medical homes have led to better care, decreased costs, and more satisfaction for providers and patients [https://patientsmedicalhome.ca/, 97]. Other positive aspects of the patient’s medical home, aside from increased continuity of care and the availability of additional health care services, may lie within the aspect of time spent together [98] or improved disease progression [99], which is also addressed within the patient’s medical homes.

### Strengths and limitations

The strength of this critical review lies in the systematic search approach, which only included papers that operationalized or specifically described trust. Despite this approach, we cannot ensure that we have included all empirical contributors to patient-physician trust that have been researched. While the systematic search did limit bias in the identified contributors within the critical assessment of what could be modifiable or not, the critical assessment could be biased through the author’s background. However, we discussed the process in depth as a team.

Our search strategy included psychological safety as a synonym for trust, as well as the terms rapport, alliance, and relationship. We checked indexed search terms to ensure the inclusion of relevant synonyms. In the past, trust was more conceptualized as rapport or alliance, whereas today, it is associated with a newer term: “psychological safety.” While we tried to include relevant search terms, we might have missed some, limiting the results.

While our search was not limited to patients trusting their physicians, most papers focused on this and excluded physicians’ trust in their patients. Dyadic analyses of patient-physician trust are scarce. However, Petrocchi et al. (2019) have begun investigating patient-physician trust as a dyad [100]. Some papers only reported correlations of trust with unmodifiable, less relevant, but easy-to-gather factors, such as sex or age. Thus, more contributors to trust may have yet to be investigated.

### Implications for future research

Interestingly, many non-modifiable or insignificant contributors, such as physician or patient demographics, were investigated in almost every study we reviewed. However, the most promising contributors, such as health education, were barely explored. Future research should investigate modifiable and promising contributors to a trusting relationship that have, as yet, been barely researched, including patient psychological factors and additional healthcare services. Additionally, factors that have not been investigated should be addressed, including digitized healthcare settings and how telemedicine, chatbots, and video consultations affect patients’ trust in physicians. Further research should also focus on measuring how successful physician interventions are, as previous research and interventions have not increased patient trust [101, 102]. Future interventions should also consider multiple contributors to trust, as they are all related. For such interventions, the outcomes for each contributor should be evaluated first, with trust as a secondary outcome.

As the present review aimed to create a model of patient-physician trust, only studies that included trust between patients and physicians were included, with other healthcare professionals excluded. However, research has already acknowledged the importance of trusting relationships for all healthcare professionals [103], which should be further expanded. Thus, shared contributors to trust between healthcare professionals, their differences, and potential leverage points should also be identified.

### Implications for practice

Our critical review has demonstrated that there are more contributors to a trusting patient-physician relationship than the theory of interpersonal trust proposes, and the context in which the patient-physician relationship takes place is highly relevant. One way to increase trust within the patient-physician relationship is to implement healthcare systems that are organized similarly to the Canadian ‘patient’s medical homes’ model. Changing the healthcare system is also an effective tool to simultaneously address multiple contributors to trust.

At the level of the institution, enhancing trust should focus on health education, which can be addressed through the implementation of self-help and support groups, providing high-quality health educational material, and training healthcare professionals.

At the physician level, we recommend taking as much time as possible for each patient to explore their perspective and current situation, organize (as much as possible) continuity of care, and ensure patient health education.

## Conclusion

Using a systematic search, our model summarizes identified modifiable contributors to a trusting patient-physician relationship. Providing sufficient time during patient-physician encounters, ensuring continuity of care, and fostering health education are promising leverage points for improving trust between patients and physicians. Future research should evaluate the effectiveness of interventions that address multiple modifiable contributors to a trusting patient-physician relationship.

### Electronic supplementary material

Below is the link to the electronic supplementary material.

Supplementary Material 1

## Data Availability

The data (review search) of the current review are available from the corresponding author on reasonable request.
