# Supplementary material for: A model of contributors to a trusting patient-physician relationship: a critical review using a systematic search strategy
Source: BMC Prim Care. 2024 Jun 1;25:194. doi: 10.1186/s12875-024-02435-z (PMC11143600; doi:10.1186/s12875-024-02435-z)
Supplement: Supplementary file 1 — Supplementary Material 1 [file 12875_2024_2435_MOESM1_ESM.docx]

**Appendix 1**

**Overview of all papers included in the review**

| **First author/ Year/Country** | **Setting and context** | **Patients** | **Physicians** | **Study design (including statistics, data collection and analysis)** | | **Trust operational-ization** | **Positive contributor to a trusting patient-physician relationship** | **Negative contributor to a trusting patient-physician relationship** | **Tested factors with no contribution to a trusting patient-physician relationship** |
| --- | --- | --- | --- | --- | --- | --- | --- | --- | --- |
| Aloba/2014/ Nigeria (1) | A single psychiatric outpatient clinic in Nigeria | Patients were treated in the psychiatric outpatient clinic for at least a year and had no chronic medical illnesses. A diagnosis of a psychiatric condition was present in all patients | Physicians were consulting psychiatrists | A cross-sectional survey among 223 participants was conducted to validate trust in a physician scale in a Nigerian psychiatric outpatient clinic. A principal factor analysis with Varimax rotation and correlations were used | | Trust was measured using the Trust in Physician Scale | -Previous numbers of admissions | -- | -Sex of the patient  -Marital status  -Diagnostic groups  -Duration of relationship with the doctor  -Duration of illness |
| Audrain-Pontevia/2018/ France (2) | Peer-to-peer online health communities (OHC’s) in France | The surveyed users of OHC’s were between 18–67 (∅ 30). Of these participants, 23% had a chronic disease such as diabetes, asthma or Crohn’s disease | Individuals’ own physicians were not described | A cross-sectional survey was conducted to measure attitude, satisfaction, trust towards the physician, and  interpersonal trust among 512 OHC users in France. Data was analyzed using a structural analysis. | | Trust in the participants' own physician was measured in three dimensions and was adapted from Dooney & Cannon (3). For all questions, a seven-point Likert scale was used, ranging from strongly disagree to strongly agree | -Users interpersonal trust in OHC’s and the information that was provided and exchanged on the platform | -- | -- |
| Bachinger/2009/the Netherlands (4) | Outpatients in an internal medicine clinic of an academic medical center (AMC) in the Netherlands | Participants were between 19 and 88 (∅ 50) and had visited an internal medicine physician for various reasons | Internal medicine physicians who worked at an AMC | A cross sectional survey was conducted among 201 participants to validate the Dutch version of the Wake Forest Physician Trust Scale (WF-D) | | Trust in physicians was measured using a Dutch version of the WF-D. | -Age (the older the patient, the more they trusted their physician) | -- | -Sex  -Educational level  -Self-reported physical health status  -Self-reported mental health status |
| Baidya/2014/ India (5) | Individuals who had visited a physician at least once in the past five years in Tamil Nadu, a state in southern India | Individuals over 40 years (∅ 53) who had visited a physician within the last 5 years | Individuals’ own physicians were not described | A cross-sectional household survey was conducted to measure trust, sociodemographic variables, self-reported health status, basis of choice of physician, time spent with the physician, and physician variables among 112 participants. Data was analyzed using the Fisher exact test and simple linear regression | | An adaptation of the Trust in Physician Scale was used to measure trust |  | -- | -Sex  -Educational level  -Occupation  -Place of last health check up  -Physician’s practice background  -Physician’s sex  -Physician’s age  -Physician’s practice type  -Time spent with the physician |
| Becker/2008/  USA (6) | Multiple primary care teams from one health management organization (HMO) in Atlanta, GA, USA | Members of an HMO in Atlanta that had visited primary care teams and had at least one of the following conditions: diabetes, elevated lipids (but no history of advanced coronary artery disease), and low-risk adults with no major morbidities | Primary care physicians who worked at the HMO | A cross-sectional survey with 2,224 participants was conducted to measure health status, practice climate, trust in physicians, patient-activation measures, health behavior, psychosocial circumstances, and sociodemographic variables | | Trust in primary care physicians was measured with a scale ranging from 0 (no trust) to 100 (greatest trust) for several questions | -Medical condition (trust was higher for patients with diabetes and elevated lipids than for low-risk adults)  -Practice climate  -Amount of team visits (trust was higher for more visits) | -- | -- |
| Benjamins/2006/USA (7) | A sample from the general social survey (GSS) from 1998 among American citizens | Respondents to the GSS were not described. On average, the respondents reported ‘good’ health | Individuals’ own physicians were not described | The data of 1,274 participants was analyzed in regard to contributors to trust and whether religious beliefs and behavior influenced trust in physicians | | Personal trust in one’s physician was measured with seven questions on a five-point Likert scale ranging from strongly disagree to strongly agree and a don’t know option | -Age  -Sex  -Race  -Self-rated health  -Income  -Health insurance  -Presence of household income  -Religious service attendance (frequent and infrequent compared to never)  -Religious affiliation (Catholic, Jewish, mainline Protestant) | -Religious affiliation (Other) | -General trust in people  -Strength of the religious affiliation |
| Berry/2008/ USA (8) | Four family practice waiting rooms in clinics in Texas, USA | Patients were visitors of one of the four family practices | Physicians were family practitioners who were currently working at large group practices that hosted different specialties. The practices were affiliated with universities and/ or medical schools | The data of 869 patients was analyzed in a cross-sectional survey. Measures included knowledge of the patient, competency of physicians (as measured by the patient), and autonomy support. Data was analyzed with a 3-stage least squares procedure | | Trust in physicians was measured with the Primary Care  Assessment Survey (3) | -Knowledge of the patient  -Competency  -Autonomy support | -- | -- |
| Blanch-Hartigan/2019/ the Netherlands (9) | An experimental design, with videos representing a Dutch oncology setting | Former cancer patients | Physicians in the videos were oncologists | An experimental 2x2x2 design. A medical consultation was simulated, the physician was either male or female, showed high or low nonverbal uncertainty, and high or low verbal uncertainty. There were 505 participants randomly assigned to watch one of the videos. Afterwards, trust in the oncologists from the videos was measured. The data was analyzed using structural equation modeling | | Participants filled out a Trust in the Oncologist Scale | -Age of patients (older patients had higher trust) | -Nonverbal behavior of high uncertainty in the physician | -Verbal uncertainty of the physician  -Sex of the physician |
| Bonds/2004/ USA (10) | An internal medicine primary  care clinic at an academic medical center in the USA | Patients who visited their primary care physician | Primary care resident physicians (physicians in training) | A cross-sectional survey (mostly by telephone) was conducted among 217 patients, which measured patient demographics, patient health and well-being, patient-physician  relationship characteristics, a global doctor trust scale, and physician characteristics. To identify trust predictors, trust was converted into a dichotomous variable (high and low). Logistic regressions were conducted | | The Wake Forest Physician Trust Scale was used to measure trust | -Sex (female)  -Education (more than a high school degree)  -Age  - Sex of the resident (female)  -Previous care by the resident while hospitalized  -Sex concordance between resident and patient  -Trust in general with doctors at institutions  -Trust in general with nurses at institutions | -- | -Marital status  -Income  -Patient health  -Year of residency training  -Prior visits to the physician  -Race concordance between resident and patient |
| Brincks/2010/ USA (11) | Clients at drug treatment and HIV centers in southern Florida, USA | All patients were females with a diagnosis of HIV. The women were at least 18 years old and met the DSM-IV criteria for substance use within the last year. Cocaine was the primary or secondary drug of abuse | Individuals’ own physicians were not described | This study was a secondary analysis of the baseline data from a randomized controlled trial. The sample included 117 women. The multidimensional (internal, chance, powerful others) health locus of control (HLOC) questionnaire was filled out by the participants, with 581 significant others included in the study (and who also filled out the questionnaire). The data was analyzed using the multilevel modelling technique | | Trust in physicians was measured using the Trust in Physician Scale. | -Individual’s powerful others (healthcare providers) HLOC  -Family members’ powerful others HLOC  -Family members’ internal HLOC | -Family members’ chance HLOC | -- |
| Canavera/2021/USA (12) | Narrative essay covering different settings, the first in an inpatient oncology setting, the second in an ICU. Both settings were in the United States | A pediatric psychologist who experienced severe complications after tumor removal | Trust in different settings was described, one physician was a surgeon, one a resident in surgery | The author (n=1) wrote a narrative essay about her own experiences with physicians and the effect this had on her trust in physicians | | No measures, but a narrative of what influenced the writer's trust in physicians | -Shared decision making  -Competency of the physician  -Compassionate listening by the physician  -Communication skills  -Physician wanting to get to know the patient to build rapport  -Good eye contact  -Undivided attention (not looking into phones or computers)  -Compassion and respect for patients  -Patient willingness to reframe situations | -Nonverbal messages by the physician  -No eye contact  -physician appearing rushed  -No time or opportunities for questions  -Experiencing the treatment of family members and interactions with physicians in a negative way | -- |
| Cook/2004/ USA (13) | Two different family practice clinics in California, USA | Patients who visited the family practice clinics | Physicians from different specialties participated. Most physicians were specialized in family medicine. Other specialties were: cardiology, dermatology, hematology, internal medicine, neurology, ophthalmology, pediatrics, psychiatry, and radiology | Semi-structured focus groups and individual interviews with patients (36) and physicians (21). Focus groups informed the individual interviews | | Qualitative analysis of the transcribed focus groups and interviews using NVivo | -Physician caring and empathy  -Eye contact  -Body language  -Attentive or active listening  -Providing and explaining information  -Patient participation in the decision-making process  -Perceived competence of the physician  -Physician availability  —  -Sex concordance  -Age  -Culture, race, and ethnicity  -Education and occupation  -Health education of the patient | -Physicians' trust  -Hindering nonverbal behavior  -Physician unavailability  -Perception of physicians as being rushed or hurried  -Physicians' failure to provide adequate explanations  -Physicians' failure to make patients feel respected  -Patients' perceptions of physician distrust  —  -Managed-care settings | -- |
| Dehghan/2017/ Iran (14) | All participants were radio and chemotherapy patients from a single hospital in Iran | Breast cancer patients who were under chemotherapy or radiotherapy (stage 1–3, not end stage) treatment. They  received at least three treatment sessions | Individuals’ own physicians were not described | Cross-sectional descriptive study with two questionnaires among 411 patients. One questionnaire measured the patient-physician relationship and the other the health-related quality of life. To evaluate the questionnaire’s validity, exploratory factor analysis was conducted | | Trust in physicians was measured using the Patient-Physician Relationship questionnaire | -Patient information  -Patient decision making | -- | -- |
| Dong/2014/ China (15) | Outpatient clinics at three Shanghai (China) general hospitals | Randomly selected outpatients that were treated ≥2 times per year by the same physician between November 2008 & December 2008  Patients had to be 18 or older | Individuals’ own physicians were not described | There were 352 patients randomly approached while waiting to see a physician and asked to fill out a translated version of the Wake Forest Physician Trust Scale. Data was analyzed using spearman’s correlations | | Trust in the physician was measured with the C-WFPTS  (Chinese version of the Wake Forest Physician Trust Scale) | -Educational level  -Increasing age of the patient  - More physician visits | -- | -Sex  -Birthplace of the patient  -Type of insurance |
| El Malla/2012/ Egypt (16) | Children’s Cancer Hospital (CCH) in Cairo, Egypt. The CCH was a free-of-charge hospital founded by donations | All parents of children newly diagnosed with cancer and admitted to receive the first chemotherapy cycle | Individuals’ own physicians were not described | Two study-specific questionnaires were developed on the basis of in-depth-interviews. Questionnaires were filled out by 304 parents at two different time points: at the start of the first chemotherapy cycle and before the third chemotherapy cycle. For the outcome ‘trust in the physician’, the percentage of parents in each category of the independent variable was calculated. Then, the relative risk (RR) was calculated, including 95% CIs. | | Trust was measured with single items in the questionnaire at the second time point | -Caring disposition of the physician  -Conversational style of the physician (kind and thoughtful)  - Intellectual and emotional needs of the parents were met | -Disrespectful and arrogant attitude  -Giving information in an insensitive manner |  |
| Fiscella/2004/ USA (17) | The study was conducted in managed-care organizations with primary care physicians in the United States | Two standardized patients (SP’s) visited the physician unannounced. Fifty regular patients of the physicians filled out a survey | One hundred community-based primary-care physicians who were members of a local managed-care organization | SP’s visited the physicians. This visit was recorded and physician behavior was assessed afterwards. The SP’s and regular patients of the physicians filled out a survey to measure trust. Data was analyzed using multilevel modelling | | Physician trust was measured using the 8-item Primary Care Assessment Survey trust subscale | -Patient age  -Health condition of the patient  -Good mental status of the patient  -Physician age  -Longer physician-patient relationship  -Family practice specialty  -Length of the visit  -Exploring patient experience of disease and illness | -Somatization of the patient | -Sex  -Finding common ground  -Physician understanding the patient’s social context |
| Gopichandran/ 2015/India (18) | Rural and urban settings in Tamil Nadu, south India | Adults living in four districts of Tamil Nadu were chosen through a multistage sampling method | The participants answered questions about their primary care physicians. There was a  variety of physicians:  - government doctors  -private practitioners  - unqualified and unregistered practitioners | A cross-sectional household survey was conducted using a structured questionnaire and was filled out by 625 participants. The questionnaire included questions concerning factors influencing trust in  physicians. The data was analyzed using structure equation modelling | | A questionnaire specific for this study was conducted to measure trust. A structural equation model was constructed with the dimensions of trust and four factors influencing trust | -Sex of the patient (women were less trusting than men)  -Physician’s behavior (clear explanations, listening patiently, smiling)  -Communication skills of the physician  -Comfort with the physician | -- | -Shared identity  -Age of the patient  -Occupation of the patient  -Place of residence of the patient (urban/rural)  -Personal involvement (knows the family situation, knows the name of patient, treats the patient like family) |
| Gupta/2014/ USA (19) | Cardiology department in an academic tertiary care university hospital in the United States | Patients who were hospitalized with acute coronary syndrome or heart failure | -- | A cross-sectional survey was distributed among 1,232 patients. The survey included multiple scales such as ENRICHD Social Support Inventory Functional  Health Literacy in Adults. Data was analyzed using multivariate analysis | | Wake Forest Physician Trust Scale | -- | -Poor social support  -Poor Coping skills  -Low health literacy | -Race  -Marital status  -Income  -Age  -Sex  -Years of education  -Employment status |
| Hamelin/2012/ Canada (20) | A tertiary teaching hospital in Canada that involved residents, undergraduates, and graduate medical students. Patients were recruited during weekly hand clinics | Patients were visitors of hand clinics and potentially needed hand surgery | Physicians were academic hand plastic surgeons. There were surgeons, residents, and undergraduate medical students involved | In this prospective clinical study, a questionnaire was distributed among 122 patients. Professionalism, physical environment, and verbal and nonverbal communication skills were assessed. After filling out the survey, the patients had a consultation with their physician. After the consultation, they filled out another survey, including an assessment of the quality of the relationship. Data was analyzed using Chi-squared tests | | The second survey (after the consultation) included statements in regard to trust in the physician | -Physician speaking in a way the patient could understand (verbal communication skills)  -Perceived technical skills of the physician by the patient  -Physician telling the truth about the medical condition of the patient  -Physician answering all questions  -Physician offering all options for medical treatment (including non-surgical options)  -Physician giving the patient enough time to explain the reason for the visit | -- | -Sex  -Age  -Education  -Income |
| Hendren/2019/ Canada (21) | An invited commentary on trust in health care, especially in the context of competency-based medicine | Not applicable | Not applicable | Invited commentary | | Described how trust can be built and which factors are important for building trust in a patient-physician relationship and in a teacher-student-relationship | -Nonverbal communication  -Verbal communication  -Time (more trust if there was more time with the physician) | -Physician’s arrogance  -Physician’s negligence  -Physician’s lack of concern | -- |
| Hillen/2011/the Netherlands (22) | The review included studies with oncology patients | Cancer Patients | Oncologists | Empirical literature published in peer-reviewed journals between October 1988 and October 2008 was searched to conceptualize oncology patients’ trust in their physicians | | The review included studies that conceptualized trust either quantitatively or qualitatively | -Age (the older the more trust)  -Sex (woman had more trust)  -Educational level  -Physician’s perceived technical competence  -Honesty  -Patient-centered behavior  -Patient-centered communication  -Continuity of care | -Ethnicity (African Americans) | -- |
| Holwerda/2013/the Netherlands (23) | Three academic hospitals in the Netherlands | Cancer patients diagnosed with breast cancer, gastrointestinal cancer, cervical cancer or  prostatic cancer within the last three months. They had an expected survival rate of at least one year | -- | A psychologist interviewed 130 patients to assess attachment style. In total, patients gave two interviews and filled out five surveys within a year. A mediation model based on a bootstrapping method was used to examine whether trust was mediated between attachment and satisfaction or attachment and distress | | Short version of the Wake Forest Physician Trust Scale | -- | -Insecure attachment style of patients | -- |
| Kanter/2019/ USA (24) | This study was conducted to compare different states in the United States and how the health care payment system works i.e., if the information is publicly accessible or not | Adults in the United States that were 18 or older and chosen from a representative sample | Individuals’ own physicians were not described, but the participants had to name the physician they saw most frequently in the last twelve months | There were 2,180 respondents to a 2-wave survey. The first survey was distributed before payments were made public, the second survey was distributed 2 years later | | The 5-item validated Wake Forest measure of Trust in one’s own physician | -- | -Public disclosure of payments (regardless of whether  respondents knew their physicians had received payments) | -- |
| Kao/1998a/USA (25) | The study was conducted in Atlanta (United States) in a national managed-care organization | Individuals 18 or older who made at least one visit to a primary care office during the period between January 1994 and June 1995 | Primary care physicians who had at least 40 eligible plan members in their practices.  - 15 salaried  -15 fee-for-service | A cross-sectional survey was conducted and filled out by 292 patients. Questions covered whether physician choice,  length of the patient-physician relationship, and the perceived physician payment method predicted patients’ trust in their physicians. Data was analyzed using linear regression models | | A 16-item scale was used to assess patients’ trust in their physician (unpublished) | -Having enough choice of physicians  -Longer patient-physician relationships  -Trust in a managed-care organization | -- | -Having a choice of health plan  -Cynicism  -Belief in the goodness of people  -Length of treatment plan enrollment (in years)  -Number of primary care office visits  -Age  -Sex  -Race  -Self-perceived health status |
| Kao/1998b/ USA (26) | Patients from three different states in the United States with the same insurance were interviewed | Individuals 18 or older who had a primary care physician visit in 1995 and were enrolled in managed care or indemnity plans of a large, national health insurer | Primary care physicians based on their payment method | Cross-sectional telephone interview survey among 2,086 patients. They were asked about their physician’s payment and patient-centered care. T-tests were used to compare patient trust by actual and perceived method of physician payment | | A 10-item scale was developed to measure trust based on an existing trust-scale | -Physician paid by the number of office visits (instead of a fixed salary)  -Patients not knowing how the physician is paid | -- | -- |
| King/2021/ USA (27) | Hospital-based addiction medicine consultation service with comprehensive care including physicians and social workers who offered support after hospital discharge to patients identified with substance use disorder during their hospitalization | Patients with substance use disorder seen and referred to an addiction consulting service during hospitalization | Addiction medicine physicians and advance practice  providers | Two surveys were filled out by 328 patients, the first while hospitalized and the second 30–90 days after hospitalization. The questionnaire included demographic questions, and questions about substance use and patient experience. Trust trajectories were modeled using discrete mixture modeling | | Wake Forest Provider Trust Scale | -Patients receiving addiction consulting services | -- | -- |
| Kowalski/2009/ Germany (28) | All 49 breast centers in the German state of North Rhine-Westphalia | Patients who had undergone inpatient surgery for newly diagnosed breast cancer between February 1 and July 31, 2006 and had at least one malignancy as well as at least one postoperative histology. Only female patients were included | Individuals’ own physicians were oncologists | A cross-sectional study using a  standardized postal questionnaire was filled out by 3,131 patients. The survey included the Cologne Patient Questionnaire for Breast Cancer, and questions regarding the physician’s communication behavior, organizational climate, and demographic characteristics. Data was analyzed using multiple linear regression | | Trust in Physician Short Form Scale with three items | -Perceived physician accessibility  -Physician’s communication behavior  -Cancer stage  -Type of treatment | -Poor organizational climate (perceived organizational chaos) | -Perceived admittance processing |
| Kushnir/2008/ Israel (29) | Regional child development center in Israel among Israeli parents, Jews and Bedouins | Children 6 months to 6 years of age, underwent rehabilitation therapy (due to disability diagnosis) at the center at least once a month and whose parents were given instructions for home therapy and were followed by the hospital for at least six months | Five Jewish, Arab-speaking pediatricians with substantial experience working within the Bedouin culture | One hundred and ninety-three parents filled out a questionnaire. The measures included the parents’ educational levels, and number of children in the family. In addition, there were included measures of parents’ evaluations of the pediatricians’ interpersonal  communication style. Data was analyzed using hierarchical multivariate regression | | Trust in the center’s pediatricians was measured by three survey items (e.g., I trust the physician) that were previously used and piloted | -Communication styles of interest and collaboration  -Ethnicity (Bedouins had higher trust than Jews) | -- | -- |
| Mack/2020/ USA (30) | Two different pediatric cancer hospitals in different states in the United States | One parent per family; the child had to be <18 years, and 1–6 weeks out from a diagnosis of cancer at initial contact | Primary oncologists of children with cancer | One hundred and sixty-six parents of children with cancer were surveyed multiple times (first time 1–6 week after diagnosis). Questions concerned demographics, physician’s communication and sensitivity, the role parents played and wished to play in treatment, and treatment success. Bivariable and  multivariable logistic regression was used to analyzed the data | | A single item from the Trust in Physician Scale | -High quality physician communication  -Parents receiving high quality information | -Relapse | -- |
| Mainous/2001/USA & UK (31) | Several ambulatory practices in the United States and United Kingdom | Patients that had visited an ambulatory practice | General practitioners and family medicine physicians | A cross-sectional survey was filled out by 1,068 patients measuring continuity and source of care, and the importance of the continuity of care. Data was analyzed using spearman’s correlation | | Trust in Physician Scale | -Continuity of care  -Length of time with one’s regular physician  -The importance of seeing one’s regular physician each time  -Age (older = higher trust) | -- | -Country of residence |
| Marcinowicz/ 2017/Poland (32) | Two primary care surgeries in primary health care units in north-east Poland | Outpatients of primary care surgeries | Half of the patients came to see family doctors, the others came to see a trainee | Cross-sectional study using the ‘trust in physician scale’ (TIPS). The study was  survey-based  Ninety-nine patients filled out a cross-sectional survey before their consultation | | Trust in Physician Scale | -Family doctor (compared to a trainee)  - Age (younger = higher the trust) | -- | -Sex  -Education  -Place of residence  -Self-assessment of health and declared chronic disease |
| Nelms/2014/ USA (33) | Primary care clinics in the Appalachian region of Ohio, United States. This region has a high poverty rate. Eight primary care clinics (private and university based) were involved | Patients who were current smokers. The participants were currently enrolled in a tobacco-dependence  treatment program on quitting attempts and use of smoking  cessation pharmaco-therapy (Medicaid) | Individuals’ own physicians were not described | Cross-sectional study of 229 smokers who filled out a baseline survey and were interviewed a week later. The questionnaire included  demographic information, health, health care provider-  related, and smoking-related variables | | Trust in Physician Scale | -Better self-reported health | -- | -- |
| O’Malley/2002/USA (34) | The study was conducted among targeted households based on a population-based sample in the United States | Participants were female, over 40 years old, residing in Washington, D.C., and living in a census tract where at least 30% of the households had an income <200% of the 1999 poverty guidelines for a family of four | Individuals’ own physicians were not described | A telephone survey was conducted with 1,205 individuals. They were asked about continuity of care, accessibility, and comprehensive service delivery.  Data was analyzed using the Chi-squared test. | | Trust was measured with a single item asked by phone (altogether, how much do you trust your doctor? On a scale from 0 to 10, whereas 0 means not at all and 10 means completely) | -Continuity with a single clinician  -Organizational accessibility of the practice  -Comprehensive care  -Coordination of specialty care services  -Age (the older the more trust)  -Health (the healthier the more trust)  -Insurance status | -More formal education | -Geographic or financial access |
| Oguro/2021/  Japan (35) | An out-sourced company recruited a desired number of patients with non-communicable diseases through an online-tool in Japan | Japanese individuals aged 20 years or older with non-communicable diseases such as cardiac disease,  diabetes, cancer, depression, and rheumatic disease | -- | A cross-sectional online survey was filled out by 661 patients. Measurements were for general levels of interpersonal trust and satisfaction. Data was analyzed using explanatory factor analysis. | | Japanese version of the Abbreviated Wake Forest Physician Trust Scale | - Age (older = higher trust) | -Dissatisfaction with a family member’s medical care  -Graduate school education (lower trust than those with junior high school education) | -Duration of the relationship with the physician |
| Parchman/2004/USA (36) | A longitudinal survey sample of a Medicare population | Participants were 65 and older | Individuals’ own physician were not described | Data from 10,323 participants was analyzed. Questions concerned the length of the relationship with a physician, communication, and the delivery of preventive services. Data was analyzed using path analysis and regression analysis | | Unclear | -Preventive service delivery  -Length of the physician-patient relationship (communication, accumulated knowledge of the patient by the physician) | -- | -- |
| Rawaf/2007/ USA (37) | Three urban tertiary medical centers and their primary care departments in the United States | White and African-American patients diagnosed with hypertension | Primary care physicians | In a cross-sectional survey among 793 patients, sociodemographic questions, prior experiences with providers, and blood pressure control questions were asked | | An 11-item scale adapted from a validated Trust in Physician Scale | -Race (white individuals)  -Employed  -Higher level of education  -Experiences with the provider (more information/ exchange about hypertension and its management) | -- | -- |
| Shaya/2019/ Lebanon (38) | Twenty-seven individuals from different governorates and residential areas (urban, suburban and rural) in Lebanon. The study was conducted in the context of the Lebanese health care system | The Lebanese public (not only patients) could participate in the study (convenience and maximum variation sampling methods) | Not applicable | Exploratory qualitative  design with semi-structured interviews | | The interview data was analyzed with a grounded theory methodology that resulted in a framework describing factors influencing trust in physicians | -Country of physician training (North America/Western Europe)  -Institution of practice (reputable)  -Years of experience (more)  -Being experienced vs. being up-to-date (up-to-date)  -Physician’s attire and hygiene (professional attire)  -Physician being competent  -Good rapport  -Encounter time (longer)  -Not money oriented  -Physician being recommended by a family member  -Physician being a family member  -Physician being featured in media  -Good reputation of the physician | -Country of physician training (eastern Europe)  -Institution of practice (non-reputable)  -Physician’s attire and hygiene (unprofessional attire)  -Physician being incompetent  -Making major mistakes (e.g., leading to death)  -Poor rapport  -Encounter time (shorter)  -Free consultation/ medication (suspicious about intention)  -Money oriented  -Poor reputation of the physician | -Physician’s sex  -Not making mistakes (small ones)  -Educating patients  -Consultation fees |
| Shoemaker/ 2019/USA (39) | Most recent childbirth (if there was more than one) | Participants were 314 women over the age of 18, living in the United States, who had given birth to at least one child | Individuals’ own physicians were not described | An online survey with four validated sub-surveys was distributed. Next to trust, patient-level collaboration, adverse events, and institutional betrayal was measured. Data was analyzed with correlation analysis and mediation models | | Trust in Physicians and Medical Institutions Scale | -Patient-physician  alliance in decision-making | -Experiences of adverse events  -Unexpected diagnoses  -Unexpected procedures  -Institutional betrayal | -- |
| Thom/1997/ USA (40) | Three diverse settings in the San Francisco Bay Area (United States). Setting one was a university-based family practice. Setting two was a sample of English-speaking Hispanic women who had visited a family practice residency clinic in San Jose within the last six months. The third setting consisted of participants who were recruited in a publicly supported medical clinic in a lower income area | The 29 participants were highly diverse (recruitment in different settings) and between 23 and 72 years old | Individuals’ own physicians were not described | Four semi-structured focus groups with 4 to 11 members were conducted, audio-recorded, transcribed, and coded. Data was analyzed using the techniques of grounded theory | | The working definition of trust was “the patient’s  confidence that the physician will do what is best for  the patient.” | -Thoroughly evaluating problems  -Understanding the patient's individual experience  -Expressing care  -Providing appropriate and effective treatment  -Communicating clearly and completely  -Building a partnership/sharing power  -Demonstrating honesty/respect for the patient  -Predisposing factors  -Structural/staffing | -- | -- |
| Wang/2018/ China (41) | Twelve leading public hospitals (in terms of patient volume) in China from different areas and provinces | There were 5,714 inpatient and outpatients | Individuals’ own physicians were not described | A survey was distributed in the hospitals. Variables included: age, sex, ethnicity, marital status, educational level, occupation, local/nonlocal residence,  urban/rural residence, insurance type, personal monthly income, religious beliefs, family economic status, whether the respondent was a first-time  visitor to the hospital, overall satisfaction with life,  perceived importance of personal health, general level of optimism/ pessimism, and inpatient or outpatient status. Data was analyzed with one-way ANOVAs and mixed-effects regression models | Chinese version  of Wake Forest Physician Trust Scale. | | -Inpatient setting (compared to outpatient)  - Family economic status | -Sex (men)  -Previous visits to the hospital  -Departments (medical, surgical, pediatrics, gynecology, and obstetrics)  -Age  -Education  -Personal monthly income  -Overall satisfaction with  current life status  -Degree of emphasis on  personal health | -Occupation of the patient  -Household registration (urban vs. rural)  -Basic medical insurance for urban employees  -New rural cooperative medical system  -Commercial medical insurance |
| Weng/2008/ Taiwan (42) | One hospital in Taiwan | There were 983 patients that had visited a hospital in Taiwan | Thirty-nine physicians of the 983 patients. Their specialty was either surgery or internal medicine | Nurse practitioners conducted structured interviews with patients to measure trust, the patient-physician relationship, and satisfaction with the physician. Physicians were also asked about the patient-physician relationship. Three nursing directors were used as an external source to assess the emotional intelligence of physicians. Path analysis was used to analyze the data | | Trust in the Medical Profession Scale | -Physician’s emotional intelligence  -Higher rates of patient follow-up visits | -- | -Age of the physician |
| Wolfson/2021/USA (43) | This commentary was written as part of the Building Trust initiative of the American Board of Internal Medicine Foundation | Not applicable | Not applicable | Participants of the trust practices network (a branch of the Building Trust initiative) offered examples of how they have built trust. These contributions led to four potential factors that increased trust in healthcare | | -- | -Competency  -Caring (compassion, empathy, and concern for the patient’s welfare)  -Communication (respect and understanding for the patient)  -Comfort | -- | -- |
| Wu/2021/China (44) | Hospitals (103) in different areas of China (developed and undeveloped regions) | There were 2,256 mandarin speaking patients. The mean age was 43.2 years (SD = 15.9), ranging from 18 to 95 years | Individuals’ own physicians were not described | A cross-sectional survey was distributed to patients. Measures included demographic characteristics, the Consultation and Relational Empathy Scale, and the Chinese version of the Patient-Doctor Relationship Questionnaire. The data was analyzed using Pearson's correlation and Hayes mediation analysis | | The Chinese version of the Wake Forest Physician Trust Scale | -Physician’s empathy  -Patient’s overall trust (propensity to trust) | -- | -- |
| Yang/2021/ China (45) | The study was conducted in a single tertiary hospital in suburban China within four departments (Emergency Medicine,  Pediatrics,  Cardiology, and  Orthopedics) | Patients in outpatient clinics of emergency medicine, pediatrics, cardiology, and orthopedic surgery. In pediatrics, parents were surveyed if the patient was under than 18 years of age | Different physician specialties such as emergency medicine, cardiology, pediatrics, and orthopedics | A cross-sectional survey that included measures about demographics, educational level, socioeconomic status, insurance type, and questions regarding patients’ perceptions of care. The survey was filled out by 436 patients who had previously seen their physician. Data was analyzed using correlations and ANOVA’s | | Chinese version of the Wake Forest Physician Trust Scale | -Trust in emergency physicians and cardiologists was higher | -Lack of improvement in condition  -Trust in pediatricians was lower  -Dissatisfaction with the hospital in general | -- |
| Zhao/2016/ China (46) | Two tertiary, public hospitals in China | Outpatients that had visited and were registered /admitted to one of the hospitals | Individuals’ own physicians were not described | A cross-sectional survey was filled out by 100 patients and included questions about demographics and insurance. Data was analyzed using a binominal logistic regression | | Trust was measured using a 10-item Likert scale | -Age of patients (older = higher trust)  -Annual income of patients (higher income = higher trust)  -Education level of patients (higher education = higher trust)  -Type of health insurance coverage (coverage = higher trust) | -- | -Sex  -Marital status  -Household registration |

1. Aloba O, Mapayi B, Akinsulore S, Ukpong D, Fatoye O. Trust in Physician Scale: Factor structure, reliability, validity and correlates of trust in a sample of Nigerian psychiatric outpatients. Asian Journal of Psychiatry. 2014 01 Oct;11:20-7.

2. Audrain-Pontevia AF, Menvielle L. EFFECTS of INTERPERSONAL TRUST among USERS of ONLINE HEALTH COMMUNITIES on PATIENT TRUST in and SATISFACTION with THEIR PHYSICIAN. International Journal of Technology Assessment in Health Care. 2018;34(1):56-62.

3. Safran DG, Kosinski M, Tarlov AR, Rogers WH, Taira DH, Lieberman N, et al. The Primary Care Assessment Survey: tests of data quality and measurement performance. Medical care. 1998;36 5:728-39.

4. Bachinger SM, Kolk AM, Smets EM. Patients' trust in their physician--psychometric properties of the Dutch version of the "Wake Forest Physician Trust Scale". Patient Education & Counseling. 2009 Jul;76(1):126-31.

5. Baidya M, Gopichandran V, Kosalram K. Patient-physician trust among adults of rural Tamil Nadu: A community-based survey. Journal of Postgraduate Medicine. 2014 January-March;60(1):21-6.

6. Becker ER, Roblin DW. Translating primary care practice climate into patient activation: The role of patient trust in physician. Medical Care. 2008 August;46(8):795-805.

7. Benjamins MR. Religious influences on trust in physicians and the health care system. International Journal of Psychiatry in Medicine. 2006;36(1):69-83.

8. Berry LL, Parish JT, Janakiraman R, Ogburn-Russell L, Couchman GR, Rayburn WL, et al. Patients' commitment to their primary physician and why it matters. Annals of Family Medicine. 2008 January/February;6(1):6-13.

9. Blanch-Hartigan D, van Eeden M, Verdam MGE, Han PKJ, Smets EMA, Hillen MA. Effects of communication about uncertainty and oncologist gender on the physician-patient relationship. Patient Education and Counseling. 2019 September;102(9):1613-20.

10. Bonds DE, Foley KL, Dugan E, Hall MA, Extrom P. An exploration of patients' trust in physicians in training. Journal of Health Care for the Poor and Underserved. 2004 May;15(2):294-306.

11. Brincks AM, Feaster DJ, Burns MJ, Mitrani VB. The influence of health locus of control on the patient-provider relationship. Psychology, health & medicine. 2010 Dec;15(6):720-8.

12. Canavera K. Rebuilding trust. Patient Education and Counseling. 2021 May;104(5):996-7.

13. Cook KS, Kramer RM, Thom DH, Stepanikova I, Mollborn SB, Cooper RM. Trust and Distrust in Patient-Physician Relationships: Perceived Determinants of High- and Low-Trust Relationships in Managed-Care Settings. [References]: Kramer, Roderick M [Ed]; Cook, Karen S [Ed]. (2004). Trust and distrust in organizations: Dilemmas and approaches. (pp. 65-98). xii, 381 pp. New York, NY, US: Russell Sage Foundation; US.; 2004.

14. Dehghan H, Keshtkaran A, Ahmadloo N, Bagheri Z, Hatam N. Patient Involvement in Care and Breast Cancer Patients' Quality of Life- a Structural Equation Modeling (SEM) Approach. Asian Pacific journal of cancer prevention : APJCP. 2018 26 Sep;19(9):2511-7.

15. Dong E, Liang Y, Liu W, Du X, Bao Y, Du Z, et al. Construction and validation of a preliminary Chinese version of the Wake Forest Physician Trust Scale. Medical Science Monitor. 2014 05 Jul;20:1142-50.

16. El Malla H, Kreicbergs U, Steineck G, Wilderang U, El Sayed Elborai Y, Ylitalo N. Parental trust in health care - A prospective study from the Children's Cancer Hospital in Egypt. Psycho-Oncology. 2013 March;22(3):548-54.

17. Fiscella K, Meldrum S, Franks P, Shields CG, Duberstein P, McDaniel SH, et al. Patient trust: is it related to patient-centered behavior of primary care physicians? Medical Care. 2004 Nov;42(11):1049-55.

18. Gopichandran V, Chetlapalli SK. Trust in the physician-patient relationship in developing healthcare settings: a quantitative exploration. Indian journal of medical ethics. 2015 01 Jul;12(3):141-8.

19. Gupta C, Bell SP, Schildcrout JS, Fletcher S, Goggins KM, Kripalani S. Predictors of health care system and physician distrust in hospitalized cardiac patients. Journal of health communication. 2014;19(Supplement 2):44-60.

20. Hamelin ND, Nikolis A, Armano J, Harris PG, Brutus JP. Evaluation of factors influencing confidence and trust in the patient-physician relationship: A survey of patient in a hand clinic. Chirurgie de la Main. 2012 April;31(2):83-90.

21. Hendren EM, Kumagai AK. A Matter of Trust. Academic medicine : journal of the Association of American Medical Colleges. 2019 01 Sep;94(9):1270-2.

22. Hillen MA, De Haes HCJM, Smets EMA. Cancer patients' trust in their physician - A review. Psycho-Oncology. 2011 March;20(3):227-41.

23. Holwerda N, Sanderman R, Pool G, Hinnen C, Langendijk JA, Bemelman WA, et al. Do patients trust their physician? the role of attachment style in the patient-physician relationship within one year after a cancer diagnosis. Acta Oncologica. 2013 January;52(1):110-7.

24. Kanter GP, Carpenter D, Lehmann LS, Mello MM. US Nationwide Disclosure of Industry Payments and Public Trust in Physicians. JAMA network open. 2019 05 Apr;2(4):e191947.

25. Kao AC, Green DC, Davis NA, Koplan JP, Cleary PD. Patients' trust in their physicians: Effects of choice, continuity, and payment method. Journal of General Internal Medicine. 1998;13(10):681-6.

26. Kao AC, Green DC, Zaslavsky AM, Koplan JP, Cleary PD. The relationship between method of physician payment and patient trust. Journal of the American Medical Association. 1998 18 Nov;280(19):1708-14.

27. King C, Collins D, Patten A, Nicolaidis C, Englander H. Trust in Hospital Physicians Among Patients With Substance Use Disorder Referred to an Addiction Consult Service: A Mixed-methods Study. Journal of addiction medicine. 2021;09.

28. Kowalski C, Nitzsche A, Scheibler F, Steffen P, Albert U-S, Pfaff H. Breast cancer patients' trust in physicians: The impact of patients' perception of physicians' communication behaviors and hospital organizational climate. [References]: Patient Education and Counseling. Vol.77(3), 2009, pp. 344-348.; 2009.

29. Kushnir T, Bachner YG, Carmel S, Flusser H, Galil A. Pediatricians' communication styles as correlates of global trust among jewish and bedouin parents of disabled children. Journal of Developmental and Behavioral Pediatrics. 2008 February;29(1):18-25.

30. Mack JW, Kang TI. Care experiences that foster trust between parents and physicians of children with cancer. Pediatric Blood and Cancer. 2020 01 Nov;67(11) (no pagination).

31. Mainous AG, 3rd, Baker R, Love MM, Gray DP, Gill JM. Continuity of care and trust in one's physician: evidence from primary care in the United States and the United Kingdom. Family Medicine. 2001 Jan;33(1):22-7.

32. Marcinowicz L, Jamiolkowski J, Gugnowski Z, Strandberg EL, Fagerstrom C, Pawlikowska T. Evaluation of the trust in physician scale (TIPS) of primary health care patients in north-east poland: A preliminary study. Family Medicine and Primary Care Review. 2017;19(1):39-43.

33. Nelms E, Wang L, Pennell M, Wewers ME, Seiber E, Adolph MD, et al. Trust in physicians among rural Medicaid-enrolled smokers. The Journal of rural health : official journal of the American Rural Health Association and the National Rural Health Care Association. 2014 01 Mar;30(2):214-20.

34. O'Malley AS, Forrest CB. Beyond the examination room: Primary care performance and the patient-physician relationship for low-income women. Journal of General Internal Medicine. 2002;17(1):66-74.

35. Oguro N, Suzuki R, Yajima N, Sakurai K, Wakita T, Hall MA, et al. The impact that family members' health care experiences have on patients' trust in physicians. BMC health services research. 2021 19 Oct;21(1):1122.

36. Parchman ML, Burge SK. The Patient-Physician Relationship, Primary Care Attributes, and Preventive Services. Family Medicine. 2004 January;36(1):22-7.

37. Rawaf MM, Kressin NR. Exploring racial and sociodemographic trends in physician behavior, physician trust and their association with blood pressure control. Journal of the National Medical Association. 2007 November;99(11):1248-54.

38. Shaya B, Al Homsi N, Eid K, Haidar Z, Khalil A, Merheb K, et al. Factors associated with the public's trust in physicians in the context of the Lebanese healthcare system: a qualitative study. BMC health services research. 2019 27 Jul;19(1):525.

39. Shoemaker K, Smith CP. The impact of patient-physician alliance on trust following an adverse event. Patient Education and Counseling. 2019 July;102(7):1342-9.

40. Thom DH, Campbell B. Patient-physician trust: An exploratory study. Journal of Family Practice. 1997 February;44(2):169-76.

41. Wang W, Zhang H, Washburn DJ, Shi H, Chen Y, Lee S, et al. Factors Influencing Trust towards Physicians among Patients from 12 Hospitals in China. American journal of health behavior. 2018 01 Nov;42(6):19-30.

42. Weng HC. Does the physician's emotional intelligence matter?: Impacts of the physician's emotional intelligence on the trust, patient-physician relationship, and satisfaction. Health Care Management Review. 2008 October-December;33(4):280-8.

43. Wolfson DB, Lynch TJ. Increasing trust in health care. American Journal of Managed Care. 2021 December;27(12):520-2.

44. Wu Q, Jin Z, Wang P. The Relationship Between the Physician-Patient Relationship, Physician Empathy, and Patient Trust. Journal of General Internal Medicine. 2021.

45. Yang J, Lu Y, Liao X, Chang MP. Examining patient trust towards physicians between clinical departments in a Chinese hospital. PLoS ONE. 2021 November;16(11 November) (no pagination).

46. Zhao DH, Rao KQ, Zhang ZR. Patient trust in physicians: Empirical evidence from Shanghai, China. Chinese Medical Journal. 2016 05 Apr;129(7):814-8.
